# 5mC and 6mA DNA Methylation in the Fungal Kingdom: From Genome Defense to Epigenetic Regulation

**DOI:** 10.3390/epigenomes10020037

**Published:** 2026-06-05

**Authors:** Daniil P. Malyshev, Vasiliy V. Belov, Elizaveta S. Gromova, Andrey A. Eremin, Maria I. Zvereva, Alexander V. Sergeev

**Affiliations:** Faculty of Chemistry, Lomonosov Moscow State University, 119991 Moscow, Russiavasilii.belov@chemistry.msu.ru (V.V.B.); gromova@belozersky.msu.ru (E.S.G.); ereminaa@my.msu.ru (A.A.E.); zverevame@my.msu.ru (M.I.Z.)

**Keywords:** DNA methylation, 5-methylcytosine, N6-methyladenine, repeat-induced point mutation, fungal epigenomics

## Abstract

DNA methylation, the covalent addition of methyl groups to cytosine (5mC) or adenine (6mA) in DNA, is a fundamental mechanism of epigenetic inheritance conserved from bacteria to humans. Fungi provide a uniquely informative window into the evolutionary logic of methylation systems. Spanning more than 1 billion years of diversification, the kingdom encompasses species that have lost cytosine methylation entirely, lineages that use 5mC to silence transposons and drive the irreversible genome-defense process known as repeat-induced point mutation (RIP), and early-diverging lineages, in which 6mA has emerged as a prominent chromatin mark. The methyltransferases underlying these strategies (DIM-2, RID, DNMT1-RFD, DNMT5, and the MT-A70 complex) and the recently characterized demethylases Dmt1 and CcTet are structurally and mechanistically distinct from their mammalian counterparts. Here we review the mechanisms, targets, and biological functions of fungal DNA methyltransferases and demethylases, incorporating cryo-EM structural insights into DIM-2 and DNMT5 catalysis, analyses of DNMT gene loss as a continuous evolutionary process, the antiviral role of DIM-2 in vegetative hyphae, and the emerging model of 6mA as a heritable regulatory mark in early-diverging lineages. By integrating these advances, this review offers the updated and comprehensive account of DNA methylation across fungi.

## 1. Introduction

### 1.1. DNA Methylation and Epigenetic Inheritance

Methylation of heterocyclic bases in DNA transmits regulatory information across cell divisions without altering the underlying nucleotide sequence. Several chemically distinct base modifications fulfill this role in eukaryotes. The first is 5-methylcytosine (5mC)—C5-methylated cytosine and its hydroxylated derivatives. This modification is catalyzed by DNA methyltransferases (DNA MTases) using S-adenosyl-L-methionine (SAM) as the methyl donor, and has deep evolutionary roots spanning archaea to eukaryotes [1,2,3]. A second modification is N6-methyladenine (6mA). In prokaryotes, it serves multiple roles: self/non-self discrimination in restriction–modification systems, strand-discriminating mismatch repair, replication initiation timing, and virulence gene regulation [4,5]. In eukaryotes, 6mA was long regarded as absent or negligible, but has emerged in certain lineages as a functional epigenetic mark with dedicated writers, erasers, and proposed inheritance mechanisms [6].

5mC heritability rests on a two-phase mechanism proposed independently by Riggs and by Holliday & Pugh in 1975 [7,8]: de novo MTases establish the pattern on unmodified DNA, while maintenance MTases specifically remethylate the hemimethylated daughter strand after each round of replication, preserving it through cell divisions [9]. Strikingly, the same two-phase logic has now been delineated for 6mA in several early-diverging lineages: dedicated de novo enzymes establish 6mA on unmodified substrates, while distinct maintenance complexes preferentially remethylate hemimethylated ApT sites after replication [6,10,11]. How fungal DNA MTases are organized, how they evolved, and how the 6mA-based system came to dominate the genomes of early-diverging lineages are the central questions this review addresses.

### 1.2. Evolutionary Implications of 5mC

Cytosine methylation imposes a mutagenic load that shapes genome composition over evolutionary time. Spontaneous hydrolytic deamination converts 5mC to thymine rather than uracil, and the resulting T:G mismatch is repaired less efficiently than the U:G mismatch arising from unmethylated cytosine deamination [9]. Methylated CpG dinucleotides are consequently depleted over time: in humans, ~70–80% of CpG sites are methylated, and their genomic frequency is approximately five-fold below the statistically expected value [9,12]. In fungi, this effect is most strikingly manifested through repeat-induced point mutation (RIP), discussed in detail in Section 2.1 [13,14,15].

The same mutagenic pressure also explains a broader pattern: methylation of gene bodies—an ancient eukaryotic trait documented in animals and plants—was largely lost in fungi [16,17,18,19]. Since methylation of transcribed exons creates a mutational burden on coding sequences, gene body methylation may have been lost early in fungal evolution because this mutagenic cost outweighed its adaptive value, albeit with some exceptions [16,18,19]. The adaptive value of gene body methylation in fungi was also likely modest from the outset: fungal genomes are typically compact, gene-dense, and intron-poor, providing limited substrate for the regulatory roles that gene body methylation plays in the larger, intron-rich genes typical of mammals [16], and the fungal 5mC machinery instead became specialized for transposon silencing, where the fitness benefit outweighs the mutagenic cost. This evolutionary variability—a retained enzymatic apparatus with diminished function—helps explain the wide diversity of methylation systems examined in this review.

### 1.3. The Kingdom of Fungi as a Natural Experiment

Among eukaryotic kingdoms, fungi are unusual in combining an exceptionally wide range of DNA methylation strategies. Across a clade spanning more than 1 billion years of evolution, one can observe the following: a complete loss of cytosine methylation in Saccharomycotina; CpG methylation restricted to transposable element (TE)-rich regions in most Basidiomycota; cytosine methylation targeting repeats and irreversibly mutating its own recognition sites via RIP in many Ascomycota; and near-complete replacement of 5mC by 6mA as the dominant epigenomic mark in early-diverging fungi (EDF; mainly Mucoromycota, Mortierellomycota, Glomeromycota, Chytridiomycota, Blastocladiomycota, and Microsporidia) [14,18,20,21,22]. This diversity—not in the basic chemistry of methylation, but in which enzymes are used, which genomic regions they target, and what biological purposes they serve—makes fungi a tractable system for investigating the evolutionary forces shaping DNA methylation systems and the functional significance of these modifications.

**Figure 1:**
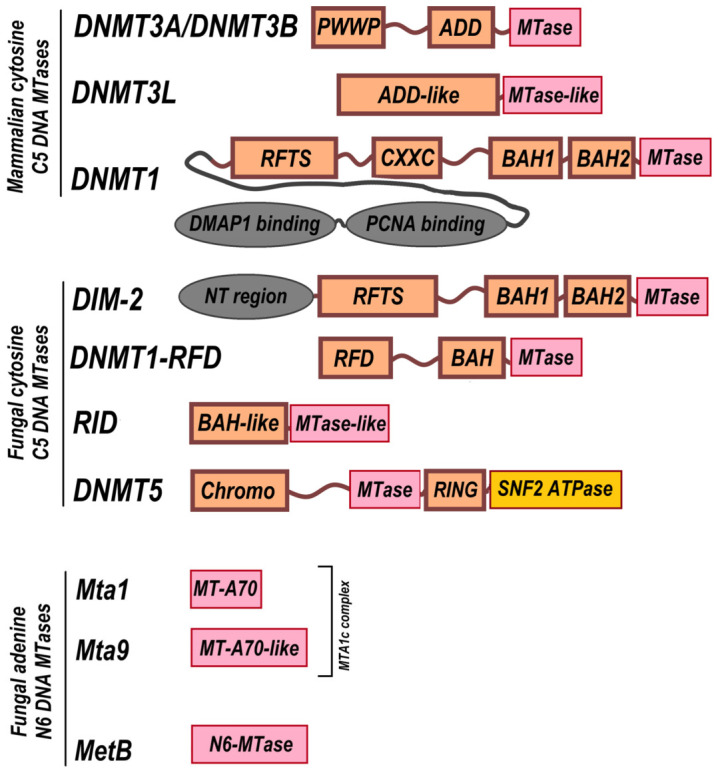
Domain architectures of DNA MTases discussed in this review. Pfam-annotated domains are shown as orange rectangles, MTase-fold domains in pink, and named functional regions without a distinct domain assignment as grey ellipses. Top row, mammalian 5mC writers: DNMT3A, DNMT3B, the catalytically inactive paralogue DNMT3L, and the maintenance MTase DNMT1 [24]. Middle row, fungal 5mC writers: DIM-2 [18,25], DNMT1-RFD [20], the Masc1/RID-subfamily enzyme RID [18,23], and the fungus- and protist-specific SNF2–MTase fusion DNMT5 [26]. Bottom row, fungal 6mA writers: the catalytic Mta1 and the inactive Mta9, which form the MT-A70 heterodimer at the core of the MTA1c complex in *Rhizopus microsporus* [22,27], and MetB, which carries a bacterial-type N6-MTase domain (Pfam PF02384) acquired by horizontal gene transfer [22]. Non-MTase complex partners (P1 of MTA1c, the HCHC complex, UHRF1) are not shown.

These strategies are implemented by a varied set of MTase families (Figure 1, Table 1). Ascomycota predominantly encode two enzymes of the DNMT1 superfamily: DIM-2 and RID. DIM-2 shares sequence similarity and domain architecture with DNMT1, including an N-terminal regulatory region with an RFTS domain flanked by BAH domains, yet functions as a chromatin-directed de novo MTase guided by H3K9me3 (histone H3 lysine 9 trimethylation), not as a maintenance enzyme [18,23]. RID belongs to the Masc1/RID subfamily (the other DNMT1-superfamily branch in Ascomycota) and initiates RIP while also nucleating the euchromatin-to-heterochromatin transition [23]. Crucially, Ascomycota lack the strict separation of dedicated maintenance and de novo activities seen in mammals; no canonical DNMT3 orthologue has been identified in fungi [18].

Basidiomycota instead use a DNMT1-class enzyme carrying a replication focus domain (DNMT1-RFD) for replication-coupled CpG maintenance methylation, as well as DNMT5—a SNF2-MTase fusion that achieves high-fidelity maintenance methylation through ATP-dependent recognition of hemimethylated sites [20,26]. A further family, DNMT2/TRDMT1, is broadly conserved but functions primarily as an RNA MTase. Histone methylation—particularly H3K9me3 and H3K4me3—provides a chromatin context that recruits or excludes some of these enzymes; this interaction is discussed mechanistically in Section 2 and reviewed in detail elsewhere [28].

A fundamentally different methylation system operates in early-diverging fungal lineages, where 6mA is established by the MT-A70 complex (MTA1c) and accumulates around the transcription start sites of expressed genes—a mechanism described in Section 4 [22,29,30].

Across these families, structural conservation varies sharply. The DNMT1 maintenance fold and the broadly conserved DNMT2/TRDMT1 framework are deeply conserved across eukaryotic kingdoms and trace back to bacterial ancestors via independent prokaryote-to-eukaryote transfers [3,18]. The MT-A70 fold, which underlies both the fungal 6mA writer MTA1c and the mammalian 6mA RNA MTase METTL3, was likely already present in the last eukaryotic common ancestor [30]. In contrast, RID is restricted to fungi (and derived within the DNMT1 superfamily, with RID evolving prior to DIM-2 [18]), DNMT5 is found only in fungi and certain protists [31], and the bacterial N6-MTase MetB entered Mucoromycota and Glomeromycota through horizontal gene transfer [22].

### 1.4. Recent Advances and Scope

Two recent reviews precede this work: Nai et al. surveyed the diversity of DNA MTases and methylomes across 40 fungal species [20], while He et al. focused on DNA methylation in plant-pathogenic fungi [32]. Several developments since then are covered here. High-resolution cryo-EM structures of DIM-2 and DNMT5 have advanced the mechanistic understanding of fungal cytosine methylation from genetic models to structural ones [25,26]. Intraspecific polymorphism of DNA MTases has been documented as an ongoing evolutionary process [33]. Antiviral protection mediated by DIM-2 in vegetative hyphae has been characterized [34]. Symmetric 6mA has been identified as a regulatory chromatin modification in early-diverging fungal lineages [27,30]. Finally, two complementary discoveries identified the first characterized fungal demethylases: Dmt1 (AlkB family, in vivo) and CcTet (TET family) [35,36].

With these advances in mind, this review covers the mechanisms and functions of DNA MTases and related demethylases in fungi (Table 1). RNA methylation and histone modifications are discussed only where they directly affect the recruitment or activity of DNA MTases. Biotechnological applications of DNA MTase inhibitors and comparative inter-kingdom methylomics are beyond its scope. The aim is to identify correlations, contradictions, and gaps in our understanding of fungal DNA MTase function.

## 2. Cytosine Methylation: Mechanisms and Functions

### 2.1. Transposon Silencing, RIP, and the De Novo DIM-2 Methylation Cascade

**Figure 2:**
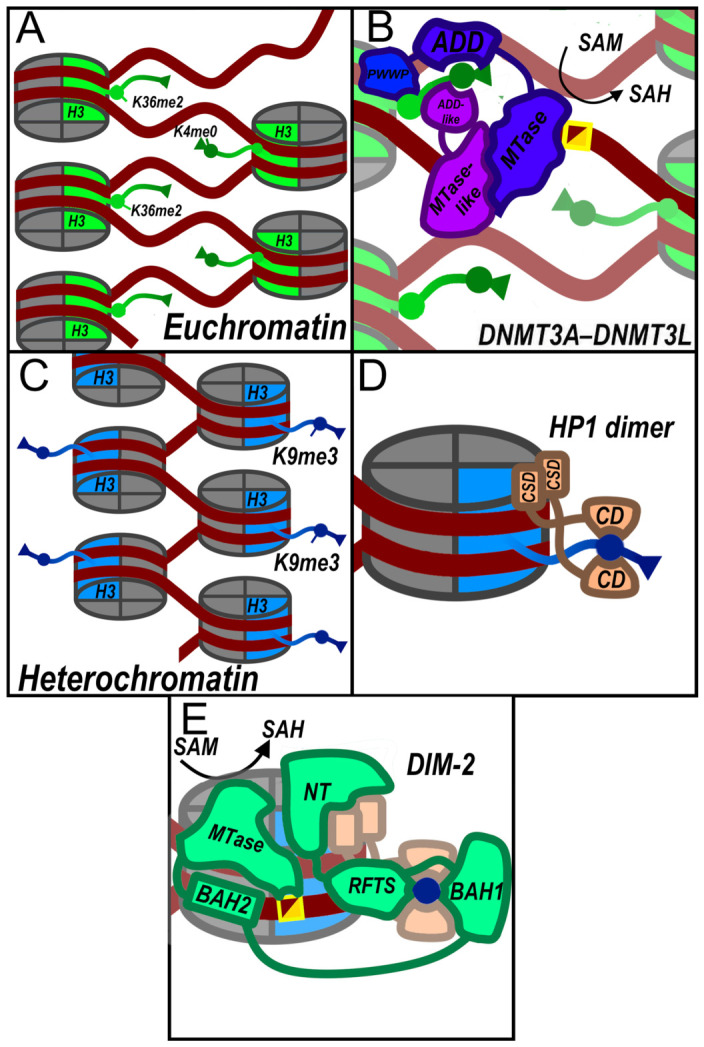
De novo cytosine methylation pathways in higher eukaryotes and fungi. (**A**) Schematic representation of euchromatin. H3 histones carrying H3K36me2 modifications and unmodified H3K4 residues (green). (**B**) Schematic of the human DNMT3A–DNMT3L complex on a nucleosome, based on the cryo-EM structures of the assembly on nucleosomal chromatin [24,41]. DNMT3A (blue) and DNMT3L (magenta) form the catalytic complex; the methylated CpG site is indicated by a half-filled yellow square. The PWWP domain of DNMT3A engages H3K36me2 on the flanking nucleosome, while the ADD domains of both DNMT3A and DNMT3L bind the unmodified H3K4 residue, permitting active complex assembly; in the absence of H3K36 methylation, PWWP engagement is markedly reduced [41]. The catalytic domain of DNMT3A methylates linker DNA using SAM as a cofactor, and the CD-like domain of DNMT3L assists in positioning the complex and serves as an additional histone-modification sensor through its ADD domain [41]. (**C**) Schematic representation of heterochromatin. H3 histones carrying H3K9me3 modifications (blue). (**D**) First stage of DIM-2 de novo methylation. An HP1 dimer (pink) with chromodomain (CD) and chromo shadow domain (CSD) assembles on heterochromatin via CD association with H3K9me3; CSDs mediate homodimerization. (**E**) Second stage of DIM-2 de novo methylation. DIM-2 domains (cyan); methylated DNA indicated by a half-filled yellow square. The NT domain of DIM-2 engages the HP1 chromo shadow domain dimer, organizing the complex. The RFTS and BAH1 domains recognize H3K9me3, while the second Bromo-Adjacent Homology domain (BAH2) contacts the flanking dsDNA. The catalytic domain methylates the target cytosine using SAM as a cofactor [25].

In fungi with an active cytosine methylation apparatus, transposable elements are transcriptionally silenced, and this silencing extends to neighboring genes. In species lacking DNA MTases, this effect is absent, indicating a direct causal relationship between methylation capacity and TE-mediated gene regulation [37]. In Ascomycota, cytosine methylation is deposited into TE-rich heterochromatin (Figure 2) via a stepwise cascade first characterized in *Neurospora crassa* through genetic screens for methylation-deficient mutants. The cascade proceeds in four steps. (i) DIM-7 recruits the H3K9 MTase DIM-5, which deposits the H3K9me3 mark (Figure 2C) [38]. (ii) Heterochromatin protein-1 (HP1) binds H3K9me3 through its chromodomain (Figure 2D) [39]. (iii) HP1 then nucleates the HCHC complex (HP1, CDP-2, histone deacetylase HDA-1 and the AT-hook chromatin protein CHAP) [40]. (iv) Within this complex, the HP1 chromo shadow domain docks DIM-2, which catalyses cytosine methylation on flanking heterochromatin (Figure 2E) [39,40].

The mechanism of DIM-2 activation in the heterochromatin context was resolved by cryo-EM [25]. DIM-2 requires simultaneous interaction with HP1 and H3K9me3. HP1 binding induces a disordered-to-ordered transition in the target recognition domain required for DNA engagement, while H3K9me3, read independently by the RFTS (replication foci targeting sequence) and BAH1 (Bromo-Adjacent Homology 1) domains, allosterically stimulates catalytic activity. This mechanism ensures that DIM-2 is only active where both factors coincide (Figure 2D–E). DIM-2 mirrors mammalian DNMT3A in coupling cytosine methylation to histone mark readout, yet the two enzymes read marks of opposite valence. DIM-2 is activated by repressive H3K9me3, directing it to inactive heterochromatin, while DNMT3A reads active H3K36me2/3 via its PWWP domain, directing methylation along the bodies of transcribed genes. Active promoters, marked by H3K4me3 and H3K36me0, are largely avoided, generating the characteristic gene-body-skewed methylation pattern of mammalian genomes (Figure 2A,B) [25,41]. DIM-2 is responsible for all detectable cytosine methylation in *Neurospora* [42] and methylates predominantly in non-CG contexts, with a preference for CpT dinucleotides [33].

The biological consequence of this repeat methylation activity is repeat-induced point mutation (RIP), a premeiotic defense mechanism that detects duplicated sequences and introduces C:G→T:A substitutions. The first documented RIP case contained 267 such substitutions [43].

Over evolutionary time, RIP has eliminated all intact mobile elements from the *N. crassa* genome, leaving centromeric regions consisting entirely of RIP-mutated TE remnants [14,44]. A related process in *Ascobolus immersus*, methylation induced premeiotically (MIP), modifies repeated sequences without mutating them. It selectively targets long interspersed nuclear element (LINE) and long terminal repeat (LTR) retrotransposons above a minimum size threshold, leaving 5S rRNA and tRNA gene repeats unmethylated [45]. Mutational signatures typical of RIP (C:G→T:A) are detected in 10 fungal genomes spanning both Ascomycota and Basidiomycota, though not always attributable to the canonical RID/DIM-2 pathway [46].

The respective roles of RID and DIM-2 in this process depend on chromatin context. Newly introduced euchromatic repeats are processed by RID alone, whereas older repeats already embedded in heterochromatin require DIM-2 [23]. Beyond the canonical H3K9me3/HP1 pathway, Dicer-independent small interfering RNAs (disiRNAs) can reversibly direct DIM-2 to promoter regions independently of the heterochromatin context [47].

### 2.2. RID: Bifunctional Initiator of RIP and Heterochromatin Formation

RID belongs to the Masc1/RID subfamily of the DNMT1 superfamily [20,46,48]. The subfamily is named after *Ascobolus immersus* Masc1, its founding member, which mediates MIP [15]. Despite carrying a conserved MTase fold, purified RID protein exhibits no detectable MTase activity in vitro [49]. Moreover, RID is not required for DNA methylation in vegetative cells: DIM-2 alone accounts for all detectable cytosine methylation in vegetative *Neurospora* [42]. This is paradoxical: deletion of the RID homologue in *Podospora anserina* (PaRid) causes female sterility, and the PaRid catalytic motif is required for sexual development despite the absence of detectable MTase activity [49]. A similar pattern is seen in *A. flavus*, where the RID-like enzyme DmtA affects aflatoxin biosynthesis and other cellular processes, yet genomic DNA methylation is negligible [50,51].

**Figure 3:**
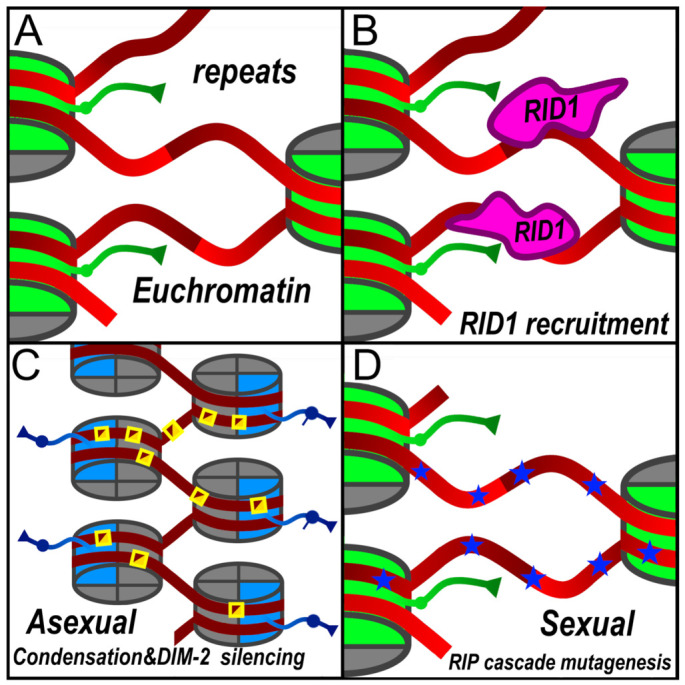
Functions of RID. (**A**) Schematic of euchromatin containing transposon repeats (red–dark red gradient). (**B**) RID recognizes DNA repeats. (**C**) In the asexual developmental pathway, RID nucleates the euchromatin-to-heterochromatin transition in an HDA-1-dependent manner; DIM-2 then catalyzes de novo cytosine methylation, silencing transposons; methylated sites are indicated by half-filled yellow squares. (**D**) In the sexual developmental pathway, RID initiates the RIP mutagenesis cascade; C-to-T transition mutations (blue stars) inactivate the repeats [52].

He et al. resolved this paradox by demonstrating that RID is a bifunctional enzyme [23]. It initiates RIP on newly introduced euchromatic repeats (Figure 3A–D) and independently catalyzes the transition from euchromatin to heterochromatin (Figure 3C), a function that does not require MTase activity. This chromatin-organizing role has not been described for mammalian DNMT3 enzymes.

These functions extend beyond chromatin organization: in *Trichoderma reesei*, both RID1 (RID ortholog) and DIM-2 participate in meiosis but at distinct stages: RID1 is required for Rad51-mediated repair of meiotic double-strand breaks, whereas DIM-2 and RID1 share a redundant function that acts upstream of Rad51 during early meiosis [52]. The double deletion of *rid1* and *dim2* causes more severe early meiotic defects than either single mutant alone, demonstrating their partially overlapping roles in meiotic progression [52]. These data collectively show that DNA MTases can perform structural roles in chromatin organization beyond direct cytosine methylation.

### 2.3. Antiviral Protection: Methylation in Vegetative Hyphae Directed by RNA Interference

In plants, RNA-directed DNA methylation requires two specialized RNA polymerases (Pol IV and Pol V) and the DNMT3-class de novo MTase DRM2 [53]. A mechanistically distinct antiviral methylation pathway operates in vegetative hyphae of *Fusarium graminearum* during infection by the gemytripvirus FgGMTV1, a circular single-stranded DNA (ssDNA) mycovirus. Small RNAs derived from the viral genome by host Dicer enzymes direct Argonaute proteins to viral promoter regions, where DIM-2 (whose activity in other contexts depends on the H3K9me3/HP1 heterochromatin cascade described in Section 2.1) is recruited to effect transcriptional silencing. This recruitment occurs without specialized RNA polymerases or the canonical H3K9me3/HP1 pathway, representing an alternative DIM-2 activity mode outside the sexual cycle [34]. The role of RNAi was confirmed genetically: deletions of *dcl1/2* or *ago1/2* accelerated viral accumulation and reduced hyphal growth rate, stress resistance, and plant infection capacity [34].

Data from *Cryphonectria parasitica* support this interpretation: both CpDmt1 (RID-like) and CpDmt2 (DIM-2-like) are required for normal antiviral responses to the Cryphonectria hypovirus, and their loss results in paradoxical spontaneous viral clearance alongside severely impaired growth and altered virulence, a self-curing phenotype never previously observed in this host–virus system [54]. Hypovirus infection independently alters promoter methylation at virulence-associated loci [55]. Taken together, these observations indicate that transcriptional silencing of foreign nucleic acids by DNA MTases is a functionally significant and likely conserved antiviral strategy in Ascomycota [34].

### 2.4. DNMT5: A High-Fidelity ATP-Dependent Methyltransferase

**Figure 4:**
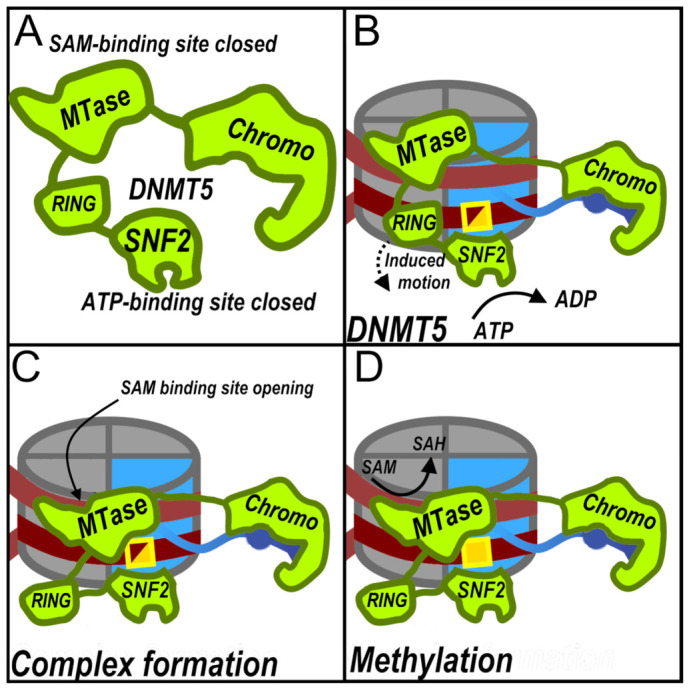
ATP-dependent maintenance methylation by DNMT5. (**A**) Free-state domain organization of DNMT5. The ATP-binding site of the SNF2 domain and the SAM-binding site of the catalytic domain are both in the closed conformation. (**B**) First stage of DNMT5 activity. The histone octamer is shown in grey, with one H3 copy highlighted in blue to mark the H3 tail engaged by the DNMT5 chromodomain. A hemimethylated CpG site (half-filled yellow square) is recognized; the chromodomain engages the H3 N-terminus. Docking of the SNF2 domain on DNA opens the ATP-binding site. ATP hydrolysis triggers a RING1 zinc finger domain movement and a protein conformational change, enabling DNA engagement by the catalytic domain. (**C**) The engagement of the catalytic domain with hemimethylated DNA opens the SAM-binding site within the catalytic domain. (**D**) Final stage: the catalytic domain methylates the target cytosine using SAM as a cofactor [26,31], generating a fully methylated CpG site (yellow square).

DNMT5 is a major CpG maintenance MTase in Basidiomycota (operating alongside DNMT1-RFD variants; see Section 2.5), distinguished from all other known DNA MTases by a fungal-specific fusion of an SNF2 ATPase domain with the MTase catalytic domain (Figure 4A); the SNF2 ATPase component is related to that of fungal Rad8 homologues, which carry the SNF2 helicase domain but lack the MTase catalytic domain [20,48]. Unlike mammalian DNMT1, which is coupled to the replication fork via proliferating cell nuclear antigen (PCNA) and the multidomain reader-ubiquitin ligase UHRF1, DNMT5 functions independently of the replication apparatus and achieves high fidelity through a fundamentally different mechanism.

Cryo-EM structures of DNMT5 from *Cryptococcus neoformans* in three conformational states revealed an unprecedented allosteric cascade [26]. Binding of a hemimethylated CpG site activates the SNF2 ATPase and promotes partial flipping of the target base out of the duplex (Figure 4B). ATP binding then reorganizes the catalytic center, permitting complete base extrusion and methylation (Figure 4C,D). Fully unmethylated DNA does not trigger this cascade and is physically expelled from the complex upon ATP binding. The result is an ATP-powered proofreading mechanism: only the correct (hemimethylated) substrate drives catalysis, while the incorrect substrate is rejected at the cost of ATP hydrolysis. An analogous mechanism has not been described for any other characterized MTase.

The functional consequences of this fidelity are particularly striking. *C. neoformans* lost its de novo MTase DnmtX 50–150 million years ago, yet has retained CpG methylation patterns through natural selection acting on rare spontaneous methylation events—the most extreme documented case of epigenetic memory in the absence of a de novo MTase [31,56].

Beyond this evolutionary persistence, DNMT5 has well-defined chromosomal functions. Within the genus *Cryptococcus*, DNMT5-dependent 5mC is required for centromere identity: native centromeres in *C. deuterogattii*—which span truncated TE remnants—are enriched for both 5mC and H3K9me2, while neocentromeres lacking both marks are unstable and predispose chromosomes to fusions and rearrangements [57]. In the rust fungus *Puccinia striiformis*, telomere-to-telomere (T2T) genome assembly revealed reduced 5mC at kinetochore attachment sites and allele-specific CpG methylation differences correlating with differential expression of virulence effector genes [58].

The relative contributions of DNMT5 and DIM-2 are nonetheless context-dependent. In *Verticillium dahliae*, DIM-2 rather than DNMT5 provides the majority of methylation under standard vegetative conditions, suggesting context-specific roles for DNMT5 that remain to be defined [59].

### 2.5. DNMT1-RFD: A CpG Maintenance MTase in Basidiomycota

**Figure 5:**
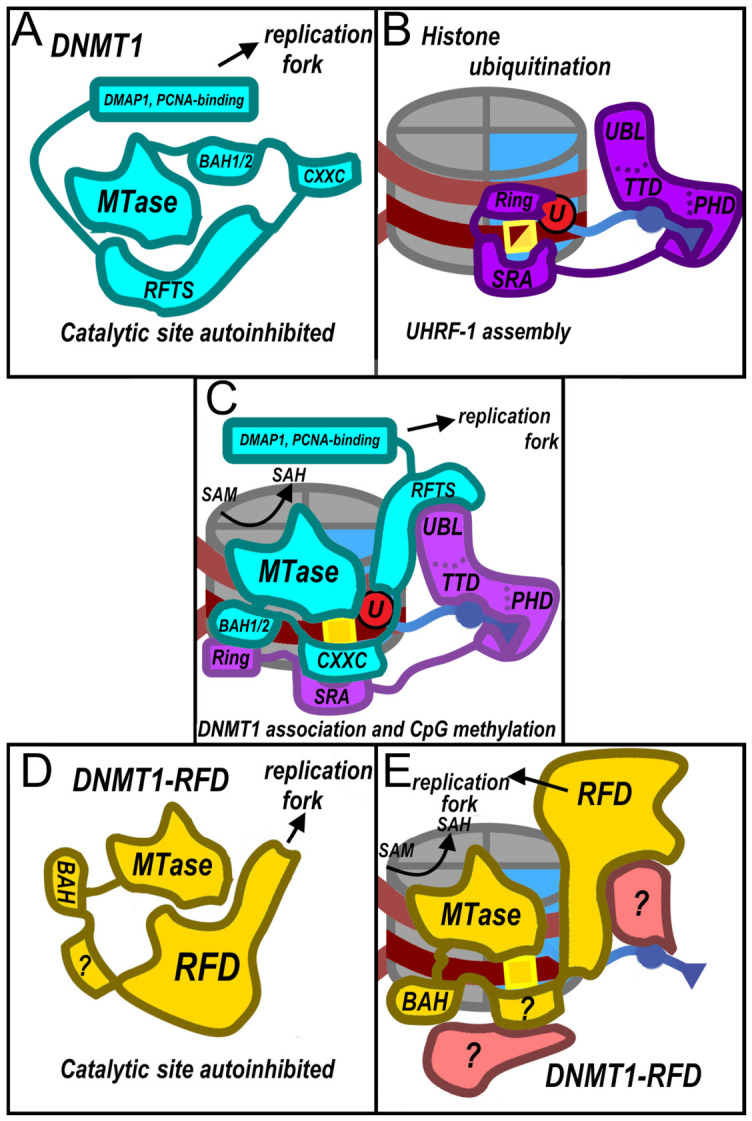
Maintenance cytosine methylation pathways in higher eukaryotes and Basidiomycota. (**A**) Domain organization of human DNMT1. The N-terminal DMAP1-binding domain and PBD/PCNA domain concentrate DNMT1 at the replication fork; the catalytic domain is autoinhibited by the RFTS in the free state [24]. (**B**) First stage of DNMT1 maintenance methylation, occurring immediately behind the replication fork. UHRF1 (magenta) is positioned at the fork in coordination with the replication machinery and engages chromatin via four reader modules: the TTD domain binds H3K9me3 on the parental nucleosome, the PHD domain contacts the unmodified H3 N-terminus, and the SRA domain flips out and recognizes the hemimethylated CpG (half-filled yellow square) on the newly replicated daughter strand. The RING domain then mono-ubiquitinates the H3 tail at K18 and K23, facilitating the recruitment and activation of DNMT1. In the schematic, “U” denotes a mono-ubiquitin moiety covalently attached to the H3 tail. (**C**) Second stage of DNMT1 maintenance methylation. DNMT1 is recruited through binding of its RFTS domain to the UBL domain of UHRF1 and the ubiquitinated histone tail; the CXXC domain engages the hemimethylated CpG, and the BAH1/2 domains contact flanking DNA. The catalytic domain flips the target cytosine out of the duplex and methylates it with SAM; fully methylated DNA is indicated by a filled yellow square [24]. (**D**) Domain organization of basidiomycete DNMT1-RFD. The RFD domain inhibits the catalytic domain in the free state and concentrates the enzyme near the replication fork. Uncharacterized domains possibly contributing to the enzymatic activity are marked with «?». (**E**) DNMT1-RFD engaged with DNA. The atomic structure of the DNMT1-RFD methylation complex has not yet been determined; possible auxiliary components («?») are shown in red. The catalytic domain methylates the target cytosine with SAM [20].

Unlike Ascomycota, which use DIM-2 and RID as the principal cytosine MTases, most Agaricomycotina employ a DNMT1-class enzyme carrying the replication focus domain (RFD) [18]. Phylogenetic analysis of 26 fungal genomes places these proteins in the DNMT1/Masc2 subgroup, sister to animal DNMT1 (Figure 5A–C) and plant MET1, and finds that DIM-2 is absent in Basidiomycota, having been lost following the divergence from Ascomycota [20,48]. Most studied Agaricomycotina harbor two to three DNMT1-RFD copies per genome: *Coprinopsis cinerea*, *Laccaria bicolor*, and *Agaricus bisporus* each encode two, *Pleurotus ostreatus* and *P. eryngii* encode three, while *Ustilago maydis* lacks any MTases and *Cryptococcus neoformans* retains only DNMT5 [16,20].

Mechanistically, the RFD discriminates between hemimethylated and unmethylated DNA at replication sites during S phase, enabling post-replicative CpG remethylation (Figure 5D,E) [20]. In the mammalian system, this domain additionally recruits DNA MTase 1 associated protein 1 (DMAP1) and histone deacetylase 2 (HDAC2) to replication foci, and has more recently been shown to read K18/K23-ubiquitylated histone H3, stimulating enzyme recruitment and catalytic activity [24]. Whether these functions are conserved in fungal homologues has not been investigated.

Functionally, the presence of RFD correlates with methylation specificity: Basidiomycota show a strong preference for CpG sites (2.76–16.4% CG, 0.38–2.47% non-CG), in contrast to the non-CG preference conferred by DIM-2 and RID in Ascomycota [16,20].

Within Basidiomycota, DNMT1-RFD operates in concert with DNMT5; together these two enzymes constitute the core toolkit for 5mC maintenance, though their respective contributions and the basis for the recurrent expansion of DNMT1-RFD copy number in Agaricomycotina genomes have not been experimentally resolved [20].

### 2.6. Methylation in Development, Pathogenesis, and Secondary Metabolism

#### 2.6.1. Developmental Regulation

In a number of fungi, 5mC methylation is coupled to developmental transitions. In *Magnaporthe oryzae*, genome-wide methylation levels differ between vegetative growth, asexual sporulation, and appressorium formation, and methylation is associated with transcriptional regulation of both TEs and developmental genes at each stage [60]. Comparable remodeling of the 5mC profile in *Arthrobotrys oligospora* affects genes involved in stimulus–response, transport, and cell division [61].

Variable MTase expression has been documented in Basidiomycota: *Ganoderma lucidum* encodes four DNA MTases with differential expression across mycelium, primordia, and fruiting bodies; in *Agaricus bisporus*, CHG/CHH methylation increases upon post-harvest browning and correlates with changes in browning-associated gene expression [62,63]. A different pattern is seen in the pathogenic yeast *Candida albicans*, where 5mC localizes not to TEs but to the bodies of genes involved in dimorphic switching and iron metabolism; methylated, transcriptionally repressed loci accumulate C→T mutations over time, consistent with the ongoing mutagenic pressure of 5mC on regulated genes [64].

#### 2.6.2. Pathogenesis

Beyond development, methylation also plays a direct role in pathogenesis. In plant-pathogenic fungi, a requirement for methylation in virulence has been documented in several systems [65].

The clearest examples come from two pathogens. In *Verticillium dahliae*, DNA methylation is required for virulence through repression of the VdRim15-dependent Ca^2+^/ROS pathway, which otherwise blocks host cell entry. In the V592 strain studied by Chen et al., VdDim2 lacks the PXSTL motif required for HP1 interaction and contributes minimally to global methylation, so the virulence-relevant MTases are VdRid and VdDnmt5, both of which are induced at the onset of infection [59,66]. In *Botrytis cinerea*, double deletion of *BcDIM2* and *BcRID2* abolishes pathogenicity entirely, making *B. cinerea* the only fungal pathogen with a demonstrated requirement for both 5mC MTases [67]. Furthermore, the 6mA MTase BcMETTL4 is also required for virulence (Section 4.2), making the methylation requirement in this species the most comprehensively documented among fungal pathogens [68].

The reverse process—active demethylation—can likewise drive infection. In *Colletotrichum gloeosporioides*, demethylation of pectinase gene promoters accompanies mango infection and directly correlates with virulence gene activation, suggesting that active demethylation can serve as a transcriptional activation mechanism during host colonization [69].

#### 2.6.3. Secondary Metabolism and Drug Resistance

DNA MTase functions are also linked to secondary metabolism, though pharmacological data require careful interpretation (Section 5.3). DIM-2 and RID co-regulate biosynthetic gene cluster (BGC) expression in *Fusarium graminearum* in a nutrient-dependent manner [70]. Conversely, DNA MTase inhibitors alter BGC expression in *Aspergillus clavatus* despite the absence of detectable genomic methylation in this species [71,72]. More broadly, epigenetic mechanisms including DNA methylation changes have been implicated in the acquisition of fungicide resistance in plant-pathogenic fungi, with differential expression of MTases and demethylases observed in response to fungicide selection [73].

### 2.7. Methylation Plasticity and Reversible Phenotypic Adaptation

#### 2.7.1. Stable Epigenetic Divergence

Clonal fungal populations, in which all individuals share the same genome, provide particularly compelling evidence that DNA methylation can drive heritable phenotypic variation without genetic change [74]. In *Cryphonectria parasitica*, a single mutation in CpBck1 (a MAPKKK orthologue) triggers genome-wide DNA hypomethylation and a morphological shift characterized by profuse, undifferentiated mycelial growth; MTase expression is suppressed in progeny, and the phenotype is stably maintained in culture without further genetic change [75].

#### 2.7.2. Reversible Methylation and Phenotypic Plasticity

In other cases, such as in plant-pathogenic fungi, methylation changes are reversible rather than fixed. *Botrytis cinerea* progressively loses virulence over eight months of laboratory culture, accompanied by methylation changes but no detectable genetic mutations; virulence and methylation are restored upon return to plant inoculation, demonstrating a causal link between methylation state and phenotype [76]. In *Fusarium graminearum*, methylation changes at more than 1000 loci—including confirmed virulence genes—accumulate over 50 subculture passages and revert upon host reinfection, emphasizing epigenetic memory of host contact [77].

Comparable reversibility has been dissected genetically in entomopathogens. The functional roles of MrRID and MrDIM-2 in *M. robertsii* were established by deletion analysis: MrDIM-2 accounts for approximately 90% of cytosine methylation, and its loss impairs radial growth, conidiation, stress tolerance, and reduces virulence (median lethal time, LT50, in *Galleria mellonella* decreased by 47.7%) [78]. Genome-wide 5mC undergoes developmental reprogramming between the mycelial (0.42%) and conidial (0.38%) stages, with 132 differentially methylated regions identified [79]. In *Metarhizium robertsii*, recovery of conidiation and virulence following host infection correlates with suppression of MrDIM-2; total methylation remains approximately constant (~0.37%), while differentially methylated regions are redistributed, indicating that redistribution rather than a change in global level underlies phenotypic plasticity [80].

Analogous subculture-associated methylation changes have been documented in *Cordyceps militaris* and in industrial strains of *Trichoderma reesei*, where methylation levels also depend on the carbon source [81,82].

Epigenetic divergence without genetic change can occur even within a single organism. In *Pleurotus ostreatus*, two compatible monokaryons with divergent subculture histories carry distinct nucleus-specific methylation profiles within the dikaryon. Reconstructed dikaryons display substantially lower global methylation than the natural strain despite sharing an identical genetic complement [83]. Independently, low-temperature stress triggers detectable methylation changes in *Pleurotus eryngii* [84]. Together, these findings show that fungal methylomes are sensitive to both long-term developmental history and acute environmental signals, generating epigenetic variation without any underlying change in DNA sequence.

## 3. Diversity and Evolutionary Dynamics

### 3.1. Methyltransferase Repertoires Across Fungal Phyla

**Figure 6:**
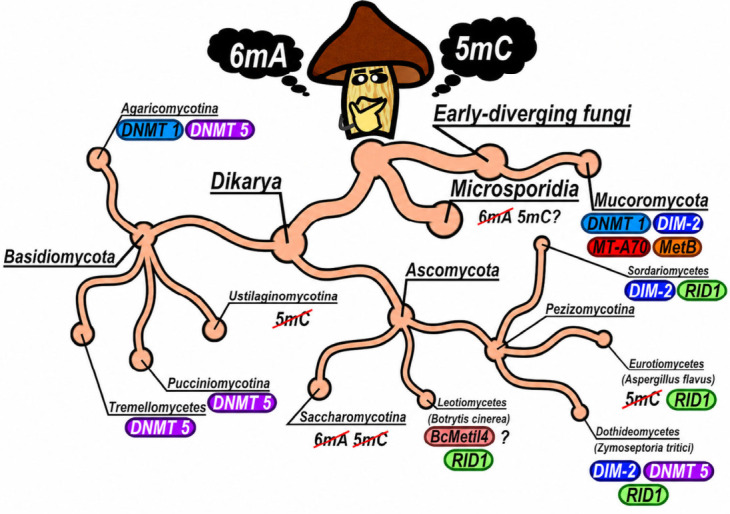
Phylogenetic distribution of DNA MTases across the fungal kingdom, summarising the survey of 528 species by Bewick et al. and later additions from Nai et al. and Lax et al. [18,20,22]. Filled icons indicate that a functional enzyme of the given family has been identified in the corresponding clade. For Microsporidia, 5mC sites have been detected by ONT sequencing in *Nosema ceranae* [85], yet no canonical 5mC MTase has been identified in this clade.

Two complementary studies provide a kingdom-wide view of DNA MTase distribution (Figure 6). Bewick et al. surveyed 5mC MTase genotypes across 528 fungal species and showed that extant fungal 5mC MTases descend from two ancestral eukaryotic lineages: a maintenance-like lineage containing DNMT1 (together with plant MET1 and CMT) and a de novo-like lineage containing DNMT3, DNMT5, and plant DRM. No canonical DNMT3 has been retained in any fungus surveyed. DIM-2 and RID arose later from within the maintenance (DNMT1) lineage, with RID evolving prior to DIM-2 [18]. Nai et al. complemented this with methylome data from 40 species, linking sequence context (CpG vs. non-CpG) to MTase genotype and documenting preferential 5mC localization at transposons at low overall levels [20].

Within this broad picture, the MTase repertoire of Ascomycota spans a continuum. Sordariomycetes (*Neurospora*, *Fusarium*, *Magnaporthe*) typically encode both DIM-2 and RID and display the full repertoire of cytosine methylation functions described in Section 2. At the opposite extreme, Saccharomycotina have lost all cytosine methylation and all detectable 5mC MTases [18,21]. Between these poles, some lineages retain only RID with minimal genomic methylation, as in *Aspergillus flavus*, where the RID-like enzyme DmtA produces measurable phenotypic effects despite undetectable 5mC [51]. Notably, a DIM-2 homologue was identified in the early-diverging Mucoromycota species *Phycomyces blakesleeanus* alongside a DNMT1-class enzyme, extending the DIM-2 family beyond Ascomycota [22].

Basidiomycota use a distinct complement of DNA MTases. The dominant enzymes are the DNMT1-class maintenance MTase carrying the RFD (DNMT1-RFD), which maintains CpG methylation at the replication fork, and DNMT5, the hybrid SNF2-MTase described in Section 2.4. Together they combine preferential CpG methylation concentrated in TE-rich regions with stage-specific expression during fungal development [20,48]. The functional significance of this CpG targeting is demonstrated by the correlation between methylation capacity and silencing of TE-flanking genes: transcriptional repression of genes adjacent to TE clusters is present in methylation-competent Basidiomycota and absent in species that have lost MTase activity, directly linking MTase genotype to genome architecture [37,86]. Early-diverging fungi have near-undetectable 5mC alongside high 6mA levels and are discussed separately in Section 4.

A noteworthy exception is the microsporidian *Nosema ceranae*. Bewick et al. found that microsporidia generally lack 5mC MTases across the 24 species examined [18]. Subsequently, Qiu et al. identified widespread CpG, CHG, and CHH methylation sites in *N. ceranae* by ONT sequencing [85]. The authors note, however, that *N. ceranae* lacks recognizable homologues of canonical MTases (DRM2, CMT3), and no functional correlation between the detected sites and gene expression was established. These findings suggest that 5mC signals are detectable in microsporidian genomes but underscore that both the enzymatic basis and functional significance of such methylation remain to be resolved.

### 3.2. Methylation Levels, TE Content, and Developmental Variation

Across the fungal kingdom, cytosine methylation levels are low: in methylation-competent species, the 5mC content is approximately 0.2–5% of cytosines. Between species, 5mC MTase genotype is the strongest predictor of methylation levels, while TE and repeat content positively correlate with 5mC and partially explains interspecific variation, with DNA transposons and LTR elements as the major contributors. Methylation is enriched at TEs relative to gene bodies in all studied species, while gene-body methylation is generally negligible [18,19]. Where TEs are abundant and methylation enzymes are present, methylation levels are highest; where TEs are scarce or MTases have been lost, methylation approaches the detection limit. This tight relationship between TE load and methylation level suggests that TE suppression is the primary evolutionary pressure maintaining cytosine methylation in the fungal kingdom.

Methylation levels are not fixed even within a single species, however. Russell et al. used isotope dilution mass spectrometry to measure 5mC content across the vegetative life cycle of *N. crassa* and found significant variation across growth stages, with an approximate inverse correlation between methylation level and transcriptional activity [87]. Between closely related species, the variation is larger and heritable. A comparative methylome study of five *Neurospora* species found 5mC content ranging from 1.3% to 2.5%, correlated with TE load. The species with the heaviest TE burden (*N. crassa*) also showed the highest TE methylation, consistent with ongoing coevolution between host and transposons [88].

Environmental context adds a third dimension of variation. In *Metarhizium anisopliae*, the 5mC content increases from approximately 0.60% under saprophytic conditions to 0.89% during simulated infection. The infection-stage methylome is enriched at secondary metabolism loci, including destruxin biosynthesis genes and the virulence gene *Mcl1*, suggesting active methylome reconfiguration at the onset of pathogenesis [89]. In *Beauveria bassiana*, ONT-based methylome analysis revealed higher 5mC levels in mycelium (4.56%) than in conidia, with differential DNA MTase expression between stages, further demonstrating that developmental stage is a primary determinant of 5mC levels in entomopathogenic fungi [90].

Not all TEs, however, are fully silenced. In *Tuber melanosporum*, a subset of TEs remains relatively hypomethylated and transcriptionally active, with higher expression in free-living mycelium than in fruiting bodies. Such a reservoir of potentially mobile elements may constitute an adaptive resource [91].

Taken together, these observations indicate that fungal methylation level is a quantitative, dynamic trait shaped jointly by TE content, developmental stage, and environmental context.

### 3.3. Intraspecific DNA MTase Polymorphism: Gene Loss as an Ongoing Process

DIM-2 is not merely a methylation enzyme: its activity promotes C→T substitutions in methylated repeats through 5mC deamination, contributing to their permanent inactivation, so its loss directly alters the mutational trajectory of TEs in the affected lineage [92]. Two organisms with documented intraspecific DNA MTase polymorphism, studied across timescales from ~10,000 years to present-day population variation, demonstrate that fungal DNA MTase gene loss is not a historical event but an active and ongoing evolutionary process.

In the wheat pathogen *Zymoseptoria tritici*, Dhillon et al. reported that *dim2* was lost through amplification-triggered RIP within the past ~10,500 years [93]. Möller et al. subsequently revised this picture: some *Z. tritici* isolates, predominantly from Iran (the species’ centre of origin), retain a fully functional *dim2* allele, indicating that inactivation is still in progress in natural populations. The mechanism of inactivation is moreover more consistent with interspecific hybridization and introgression than with the original RIP-based model. In strains that have lost functional *dim2*, DNMT5 independently maintains a residual low level of methylation, providing partial TE repression [33].

In *Magnaporthe oryzae*, the same functional outcome (loss of DIM-2 methylation activity) was achieved through at least three independent mutational events, each inactivating the gene through a distinct mechanism. The coexistence of 5mC-competent and 5mC-deficient isolates within *M. oryzae*, and the mild phenotypic impact of losing the cytosine MTase MoDMT1 under laboratory conditions, indicate that DNA MTase gene loss carries little selective pressure in certain environments [60,94]. This explains why such polymorphism can persist within a species and why gene loss proceeds as a gradual, ongoing process rather than a single ancestral event.

## 4. 6-Methyladenine: Writers, Erasers, and Functions

### 4.1. Discovery, Prevalence, and the Contamination Debate

**Figure 7:**
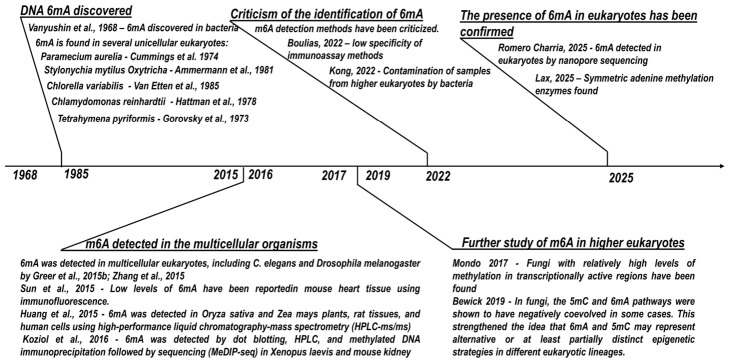
Timeline of major discoveries in eukaryotic N6-methyladenine (6mA) research [5,18,22,27,29,30,95,96,97,98,99,100,101,102,103,104,105,106].

6mA has long been regarded as a prokaryotic DNA modification (with roles in restriction–modification, mismatch repair, and gene regulation) and as an RNA modification in eukaryotes (Figure 7) [4]. Its detection in eukaryotic DNA has generated considerable interest but also serious controversy, as many early reports may have reflected methodological artifacts or bacterial contamination [5]. The problem became particularly acute when it emerged that in several groups—including insects, plants, and humans—much of the detected 6mA can be attributed to technical issues: bacterial contamination of DNA preparations, low antibody specificity, inadequate controls, and insufficiently purified samples [5,95].

Nevertheless, data from early-diverging fungi and certain other eukaryotic lineages indicate that 6mA cannot be dismissed as purely artifactual. In some fungi, 6mA reaches high levels, forming methylated adenine clusters (MAC) near transcription start sites, as validated by orthogonal methods [29]. Moreover, comparative studies indicate negative co-evolution between 6mA and 5mC, as well as a possible ancient origin of the 6mA methylation system associated with AMT1/Mta1 proteins [18,29,30].

### 4.2. Symmetric and Asymmetric 6mA

#### 4.2.1. Symmetric 6mA: The MTA1c Complex in Mucoromycota

**Figure 8:**
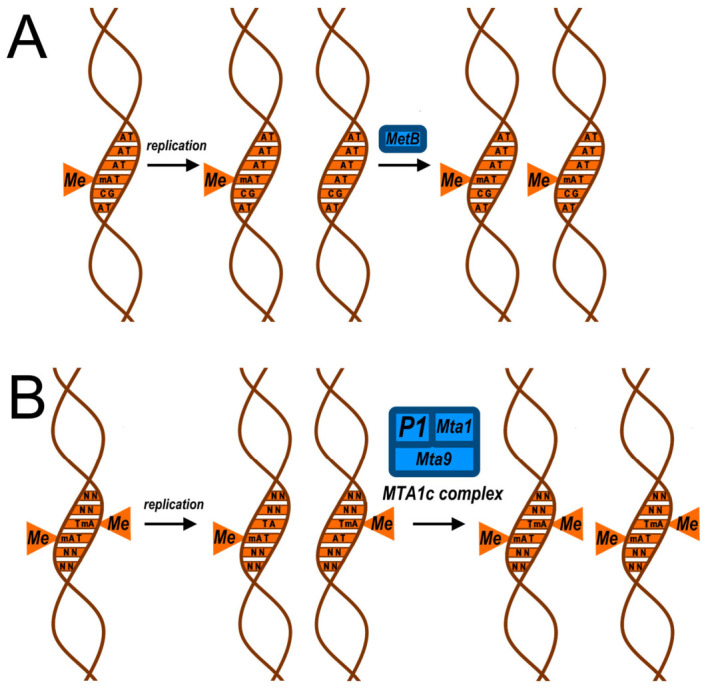
Two modes of adenine methylation in fungi, shown in the context of semi-conservative DNA replication. (**A**) the asymmetric AAA**6mA**CA motif methylated by MetB-type MTases in *Mucor lusitanicus*. Because the modification is present on only one strand and the recognition motif itself is non-palindromic, each daughter strand must be re-methylated de novo. (**B**) the symmetric ApT site methylated by the MTA1c complex (Mta1/Mta9/P1) in *Rhizopus microsporus* and by the *Tetrahymena* AMT1-containing complexes. Replication of a fully methylated ApT generates two hemimethylated daughter duplexes, each of which can be restored to the fully methylated state. Whether the MTA1c complex acts as a de novo or a maintenance MTase remains to be determined [22,68].

Lax et al. compared 6mA methylation profiles in two early-diverging Mucorales. In *Phycomyces blakesleeanus*, 91.72% of 6mA sites are symmetric, almost all occurring at ApT dinucleotides. These form MAC clusters and associate with highly expressed genes. In *Mucor lusitanicus*, by contrast, only 0.32% of sites are symmetric, and most 6mA is asymmetric and more diffusely distributed. Thus, within a single order, two distinct 6mA writing systems—symmetric and asymmetric—coexist (Figure 8) [22].

Fungi with high total 6mA levels predominantly display symmetric methylation (Figure 8, right panel). This is best characterized in *Rhizopus microsporus*. Lax et al. identified the components of the MTA1c complex in this organism—the MT-A70 family proteins Mta1 and Mta9, and the Myb-like DNA-binding protein P1. Interaction among Mta1, Mta9, and P1 was confirmed by yeast two-hybrid assay, with P1 additionally shown to be enriched at 6mA-rich chromatin regions.

In the *R. microsporus* genome, 6mA is predominantly localized at ApT dinucleotides and is mainly symmetric. It correlates with H3K4me3 and H2A.Z in open euchromatin [27]. Knockdown of *mta1* is accompanied by reduced total 6mA, a decreased proportion of symmetric sites, and altered nucleosome positioning and H3K4me3 distribution.

The mechanistic basis of MTA1c-directed methylation in *R. microsporus* is not yet fully elucidated, but the similarity of its 6mA distribution to that of *Tetrahymena thermophila* suggests a related enzymatic system [27].

#### 4.2.2. The Tetrahymena Model for 6mA Inheritance

The *Tetrahymena* system is the best-characterized model for symmetric 6mA inheritance. AMT1 was first characterized as the MT-A70 MTase responsible for all symmetric 6mA at ApT dinucleotides in *Tetrahymena thermophila*; its loss causes severe growth and developmental defects, and detection of hemimethylated sites provided initial evidence for semi-conservative 6mA inheritance [107].

Semi-conservative 6mA inheritance has been directly demonstrated in *Tetrahymena thermophila*: following replication, a fully methylated 6mApT site transiently becomes hemimethylated, and is then restored by AMT1-dependent maintenance methylation [107,108]. Wang et al. further showed that this maintenance function is carried out by two complexes sharing the AMT1 catalytic subunit: the AMT7 complex provides the principal high-processivity maintenance activity and associates with H2A.Z/H3K4me3, while the AMT6 complex is recruited by PCNA and initiates methylation restoration after replication [11]. De novo establishment of 6mA in the new macronucleus requires the MT-A70 enzymes AMT2 and AMT5 [10].

Asymmetric 6mA occurs predominantly in fungi with low overall methylation and is associated with the AAA**6mA**CA sequence motif (Figure 8, left panel). Lax et al. demonstrated that MetB is responsible for asymmetric 6mA deposition in *M. lusitanicus*: its inactivation eliminates the characteristic sequence motif and abolishes the asymmetric methylation component while preserving symmetric sites [22]. *metB* belongs to the N6-MTase family (PF02384), related to bacterial restriction MTases. Phylogenetically, the gene forms a fungal cluster consistent with ancient horizontal gene transfer to the common ancestor of Blastocladiomycota and terrestrial fungi, followed by repeated loss in Dikarya [22]. A second, independent transfer from endosymbiotic bacteria has been proposed for some Glomeromycota. Asymmetric 6mA in fungi therefore likely arose not through elaboration of an ancestral eukaryotic 6mA system, but through co-option of a bacterial MTase module.

The METTL4-like lineage warrants separate consideration. In *Botrytis cinerea*, BcMETTL4, rather than BcDAMT (a second 6mA MTase candidate), is the primary 6mA writer: inactivation of BcMETTL4 and mutation of its catalytic DPPW motif sharply reduced 6mA levels and attenuated virulence, whereas removal of BcDAMT caused neither a comparable reduction in total 6mA nor an equivalent virulence phenotype [68]. This indicates that some Dikarya may possess a distinct 6mA system evolutionarily separate from the MTA1c/metB architecture typical of EDF [68].

Regarding demethylation, at least two classes of candidate erasers have been identified in fungi. The first is the AlkB/ALKBH-like family. In *Mucor lusitanicus*, Dmt1 (a relative of C. elegans NMAD-1) reduces 6mA during the dimorphic transition. Consequently, *dmt1* mutants accumulate elevated 6mA and display impaired morphogenesis [35]. The second class comprises Ten-Eleven Translocation (TET)-like dioxygenases: in *Coprinopsis cinerea*, CcTet is the first enzyme directly characterized as a dsDNA 6mA demethylase, oxidizing 6mA to the intermediate 6hmA while retaining dual activity against 5mC [36]. Structural analysis identified Gly331 and Asp337 as critical determinants of 6mA recognition, making CcTet the first biochemically well-characterized fungal 6mA eraser [36,109].

### 4.3. Biological Functions of 6mA in Fungi

#### 4.3.1. 6mA in Transcriptional Regulation

A consistent association between 6mA and transcriptional activity has been established in several early-diverging fungi and other eukaryotes, though its functional interpretation remains debated. Mondo et al. first showed that 6mA associates with expressed genes and concentrates in dense clusters around transcription start sites (TSS) [29]. Lax et al. subsequently demonstrated that in *R. microsporus*, 6mA correlates with H3K4me3 and H2A.Z in open euchromatin regions; 6mA is underrepresented in nucleosomal DNA and predominantly localizes to linker regions, while methylated genes display more ordered nucleosome positioning. Based on these findings, the authors proposed that 6mA clusters, particularly those flanking TSSs, may promote nucleosome positioning and thereby maintain DNA accessibility for RNA polymerase II [27].

Whether this association is causal or correlative is less clear. In a comparative survey of 18 unicellular eukaryotes, Romero Charria et al. confirmed that 6mA is broadly associated with transcriptionally permissive chromatin in AMT1-encoding lineages, consistently accumulating downstream of TSSs between H3K4me3-bearing nucleosomes. However, 6mA levels changed little in many cases following transcriptional perturbation. H3K4me3 enrichment can occur without 6mA, and H3K4me3 reductions do not invariably coincide with 6mA loss. The authors therefore interpret 6mA as a mark of transcriptionally active chromatin that plays a permissive rather than instructive role in transcriptional control [30].

Building on this, Lax et al. used DAP-seq to map transcription factors at methylated genes, reconstructing a broad regulatory network through which 6mA effects extend across much of the *R. microsporus* genome. Of 58 transcription factors examined, those associated with methylated genes potentially regulate 6494 genes; the total number of genes under direct or indirect 6mA control reaches 8097—75.6% of the genome.

The functional significance of this network was confirmed through analysis of zinc metabolism and light-response pathways, implicating 6mA-dependent regulation in key adaptive and metabolic programs [110]. Thus, taken together, these studies demonstrate that DNA adenine methylation in *R. microsporus* is not a local epigenetic mark but the basis of a multi-level regulatory system that combines direct effects on chromatin structure with indirect control of transcriptional networks.

The relationship between 6mA and 5mC further clarifies this picture. In *Rhizopus microsporus*, 6mA is excluded from constitutive heterochromatin and associates with H3K4me3/H2A.Z-positive open chromatin. In *Phycomyces*, 6mA and 5mC show contrasting distributions: 5mC predominantly marks repeat-rich regions and suppresses transposon activity, while 6mA is associated with active genes and is largely excluded from 5mC-rich TE regions. In these early-diverging fungi, therefore, 6mA does not duplicate 5mC function but rather complements it [22,27].

#### 4.3.2. 6mA in Life Cycle Regulation

In the EDF *Mucor lusitanicus*, the *dmt1* gene—putatively encoding a 6mA demethylase—is involved in morphogenetic regulation: its inactivation does not block but markedly slows the yeast-to-hypha transition and impairs polar spore germination upon macrophage contact. In *Phycomyces*, the dynamics of MAC clusters correlate with transcriptional changes during both the mycelium-to-sporangiophore transition and the light response, suggesting a possible role for 6mA in asexual development and environmental sensing. In *Mucor*, loss of symmetric 6mA following deletion of *mta1* or *p1* is accompanied by growth retardation and activation of DNA repair genes, while *mta1*Δ additionally shows reduced sporulation and increased SDS sensitivity [22,35].

#### 4.3.3. 6mA in Pathogenesis

Beyond EDF, 6mA has also been implicated in pathogenesis. In *Botrytis cinerea*, MTase BcMETTL4 is a key regulator of 6mA: its inactivation reduces 6mA levels and attenuates pathogenicity in assays on tomato leaves and onion epidermis. Transcriptome analysis revealed that the *bcmettl4* mutant suppresses the expression of numerous genes bearing 6mA marks in their promoter regions; these genes are functionally associated with redox processes, secretory pathways, autophagy, and carbohydrate metabolism—all potentially relevant to pathogenesis [68].

A parallel role has been documented in mucoralean infection of animal hosts. In *Mucor lusitanicus*, the *dmt1* mutation impairs the dimorphic transition and alters spore germination following macrophage phagocytosis, indicating a possible role for 6mA in the early stages of host interaction; different components of the 6mA writing system have been shown to exert differential effects on virulence [35].

Taken together, these findings suggest that 6mA should be regarded not merely as an epigenetic mark but also as a potential regulator of pathogenicity-associated traits in certain fungi.

## 5. Methylation Detection and Functional Analysis

### 5.1. Quantification and Mapping of 5mC

Methods for detecting and mapping 5mC and its 5-hydroxymethylcytosine derivative were discussed in detail previously [111]. Whole-genome bisulfite sequencing (WGBS) is the standard approach for single-nucleotide-resolution 5mC mapping in all cytosine sequence contexts. DNA is treated with sodium bisulfite, which deaminates unmethylated cytosines to uracil (read as thymine during sequencing), leaving 5mC unchanged. In fungi, where global methylation levels (0.2–5%) can approach the bisulfite underconversion background, rigorous biological replication, spike-in control DNA, and stringent filtering are required to distinguish signal from noise [88]. WGBS has been applied to most of the species discussed in this review and remains the reference method when locus-level resolution is required.

Two mass spectrometric approaches complement WGBS by providing global quantification without base-pair resolution. Liquid chromatography–tandem mass spectrometry (LC-MS/MS) targeting 5-methyl-2′-deoxycytidine released by enzymatic hydrolysis is the method of choice when minimizing false positives is critical.

The earlier GC-MS protocol that applied acid hydrolysis to DNA preparations, cleaving the glycosidic bond and releasing free 5-methylcytosine, was unable to distinguish DNA- from RNA-derived sources. Copurified RNA was the source of the artifactual signals observed in yeast. LC-MS/MS of enzymatically fragmented DNA confirmed the complete absence of methylation in *Saccharomyces cerevisiae*, *Schizosaccharomyces pombe*, and related species with a sensitivity of 250 attomoles—below the detection limit of bisulfite sequencing [21].

More recent optimizations extend this approach. A protocol applicable to plant and fungal DNA achieves a sensitivity of 0.2 µM for HPLC-UV and 0.02 µM for HPLC-MS, with improved noise suppression and reproducibility [112].

Where single-nucleotide resolution is not required, simplified approaches are available. ddRAD-MCSeEd (double-digest restriction site-associated DNA methylation-sensitive enzyme digestion) detects differentially methylated regions at lower cost and with less DNA than WGBS, making it practical for population-scale comparisons; it has been used to track methylation changes over 50 generations of *F. graminearum* subculture [77]. Methylation-sensitive amplification polymorphism (MSAP) and ELISA-based colorimetric assays offer still higher throughput at reduced quantitative resolution; MSAP has been applied to monitor methylation under low-temperature stress in *Pleurotus* and to track virulence-associated methylation changes in *Botrytis* during cultivation [76,84]. The lower-resolution methods are appropriate for condition screening or population studies where the cost and data requirements of WGBS are not justified.

### 5.2. Detection and Mapping of 6mA

Mapping of eukaryotic 6mA has been accompanied by methodological challenges from the outset. Methylated DNA immunoprecipitation sequencing (MeDIP-seq) was used to map 6mA in *Botrytis cinerea* and its association with virulence-associated loci [68]. MeDIP-seq carries three limitations: 6mA antibodies can cross-react with other adenine modifications; substantial amounts of input DNA are required; and immunoprecipitated fragment reads cannot distinguish symmetric from asymmetric methylation—a distinction important for assessing heritability.

A further complication is contamination: Kong et al. demonstrated that much of the detected 6mA in *Drosophila*, *Arabidopsis*, and human samples reflects contamination by commensal bacteria, and that plasmids from Dam-MTase-deficient *E. coli* strains still carry sufficient residual 6mA to confound studies of endogenous enzymes [95].

These caveats underscore the difficulty of interpreting 6mA data in organisms with low endogenous levels, though they do not apply to early-diverging fungi, where methylation reaches 2.8% of adenines and has been independently confirmed by LC-MS/MS [27,29].

Single-molecule real-time (SMRT) sequencing from Pacific Biosciences avoids these limitations and is the primary technology for 6mA mapping in fungi. It detects modifications in native DNA without chemical pretreatment by measuring inter-pulse duration (IPD) kinetics as the polymerase traverses each base; 6mA produces a characteristic kinetic signature distinct from unmodified adenine. Symmetric ApT methylation is identified by simultaneous comparison of kinetic signals on both strands of the same molecule, enabling direct inference of heritability from sequencing data [22,29]. Orthogonal genome-scale validation uses HPLC-MS/MS to quantify the 6mA/A ratio, and isoschizomeric restriction enzyme pairs provide rapid qualitative confirmation: DpnI cleaves GATC only when adenine is N6-methylated, while DpnII is blocked by the same modification [22,27].

Direct nanopore sequencing (Oxford Nanopore Technologies, ONT) complements SMRT by detecting base modifications in native DNA via disruption of the ionic current signal, without bisulfite treatment or polymerase kinetics. It enables discrimination of symmetric and asymmetric methylation at single-base resolution [30]. The most technically demanding application is duplex ONT sequencing combined with Hi-C, which enabled T2T assembly of *Puccinia striiformis* and the first allele-specific methylation analysis in a fungus with spatially separated haploid nuclei [58].

Integrated chromatin profiling combines SMRT-seq (6mA), WGBS or ONT (5mC), MNase-seq, and ChIP-seq (H3K4me3, H3K9me3, H2A.Z) with DAP-seq to map relationships between 6mA, nucleosome positioning, and histone modifications at genome scale [18,27,85,107,110].

### 5.3. Genetic, Enzymatic, and Structural Approaches to Studying DNA MTase Function

#### 5.3.1. Genetic and Enzymatic Tools

The standard approach for studying methylation-phenotype relationships combines DNA MTase gene deletion—by CRISPR or homologous recombination—with genome-wide methylome mapping (WGBS or ONT) and RNA sequencing, enabling direct comparison of methylation patterns and transcriptional changes between deletion mutants and wild type. This strategy has been applied to functional characterization of DNA MTases in *Verticillium*, *Fusarium*, *Botrytis*, and several other pathogens [59,67,70]. Unbiased genetic screens in methylation-reporter strains provide a complementary approach: by selecting mutations that restore silenced reporter expression, all components of the methylation pathway can be identified, including factors not predicted by homology [39].

The cytidine analogue 5-azacytidine (5-azaC) probes 5mC-dependent phenotypes rapidly, as its incorporation into DNA sequesters and depletes DNA MTases. This approach was used to restore conidiation in phenotypically degenerate *Metarhizium* [80] and to reduce aflatoxin production in *A. flavus* [113].

However, as demonstrated by 5-azaC-induced changes in biosynthetic gene cluster expression in *A. clavatus*—a species lacking detectable genomic methylation—pharmacological results cannot be taken as evidence for the presence or functional significance of methylation without corresponding genetic deletions [72,114].

#### 5.3.2. Structural Approaches

At the structural level, cryo-EM has enabled atomic-resolution characterization of two of the most thoroughly studied fungal DNA MTases: DIM-2 activation by HP1 binding and dual H3K9me3 readout by its RFTS and BAH1 domains [25], and the ATP-dependent conformational error-correction cascade of DNMT5, which physically ejects unmethylated substrates [26]. For demethylases, the point mutation CcTet D337F has created a useful biochemical tool: the crystal structure of wild-type CcTet with 5mC-containing DNA shows that Asp337 stabilizes the 5mC substrate. The D337F mutant disrupts this interaction, selectively suppressing 5mC oxidation while preserving 6mA demethylase activity, enabling specific 6mA demethylation in vivo without affecting cytosine methylation [36,109].

## 6. Conclusions and Open Questions

In recent years, the molecular mechanisms of fungal DNA MTases have been substantially clarified. The cryo-EM structure of DIM-2 in complex with HP1, H3K9me3, and DNA revealed how chromatin mark recognition activates the enzyme [25]. For DNMT5, the ATP-dependent conformational transition that discriminates between methylated and unmethylated DNA enables maintenance of the 5mC pattern in the absence of a de novo MTase [26]. Despite these advances, the mechanism by which DNMT5 locates hemimethylated CpGs is only partly resolved: a stripped-down UHRF1 homologue assists DNMT5 in *C. neoformans*, but whether analogous adaptors operate in other fungi remains unknown [26,31].

Although the dual function of RID (RIP initiation on new euchromatic repeats and HDA-1-dependent heterochromatin condensation) has now been established genetically [23], the structural basis of its chromatin-organizing activity in the absence of detectable MTase activity remains unexplained. Cryo-EM analysis of RID in complex with its HDA-1-associated partners is the logical next step.

In the area of evolutionary dynamics and genome defense, three key findings merit emphasis. First, DNA MTase gene loss is not a discrete historical event but a continuous process, documented in *Zymoseptoria tritici* (where inactivation remains ongoing in natural populations) and as at least three independent events in *Magnaporthe oryzae* [33,93,94]. Each such loss irreversibly alters the mutational trajectory of TEs in the affected lineage.

Second, DIM-2 provides antiviral protection through an RNAi-directed mechanism in vegetative *Fusarium* hyphae, extending its functional scope beyond the sexual cycle [34]. The key open question is how fungal methylomes change during host infection or antifungal treatment. Genome-wide methylome data during active infection or drug exposure remain lacking for most species, and methylation-mediated phenotypic change may constitute a non-genetic mechanism of drug tolerance, as suggested by reversible virulence loss in *Botrytis* during laboratory culture [76]. These properties position methylation enzymes as potential targets for antifungal drug development [25,26].

Third, the 6mA system of early-diverging fungal lineages has matured from an isolated observation to a fully characterized regulatory model. MTA1c-dependent symmetric 6mA is now recognized as a bona fide epigenetic mark, likely present in the last eukaryotic common ancestor and retained as an essential modification in *Rhizopus*: its loss is lethal, and controlled reduction disrupts nucleosome positioning, histone modification patterns, and transcription [27,30].

Two demethylase discoveries have characterized the reverse process. Dmt1 in *Mucor lusitanicus* provided the first genetic evidence that a fungal enzyme reduces 6mA levels in vivo, linking this mark to dimorphism and macrophage interaction [35]. CcTet, identified in *Coprinopsis cinerea*, is the first enzyme shown to demethylate 6mA on an intact double-stranded DNA substrate. Its selective D337F variant suppresses 6mA in vivo without affecting 5mC [36,109].

These achievements raise several important questions. The situation in Dikarya remains unresolved: BcMETTL4 is known to regulate 6mA levels in *Botrytis* [68], but how widespread a comparable enzyme is across Ascomycota and Basidiomycota is unknown. It also remains unclear whether MTA1c retains DNA MTase activity in lineages that encode it but maintain low 6mA levels.

On the demethylase side, genetic validation is needed to define the precise function of CcTet in *Coprinopsis* and other Basidiomycota. Its dual substrate activity (oxidation of both 5mC and 6mA) raises the question of possible cross-regulation between these two marks through a shared demethylation mechanism. Transposon-encoded TET/J-Binding Protein (JBP) dioxygenases in Basidiomycota and Pezizomycetes add a further layer of demethylase diversity yet to be functionally characterized [115].

Underlying all these questions is a technical limitation: current sequencing technologies such as SMRT and ONT produce conflicting 6mA signals at the 0.05–0.21% levels found in Dikarya, and distinguishing genuine marks from background noise remains challenging [29,95].

Finally, no canonical 6mA reader protein has been identified in fungi. The effects of 6mA on transcription appear to operate through nucleosome positioning and direct inhibition of transcription factor binding, rather than through dedicated reader proteins [6,27,110]. The existence and identity of specific 6mA readers remains an open question in fungal epigenomics.

## Figures and Tables

**Table 1:** Overview of fungal DNA MTases and demethylases, summarising family assignment, taxonomic distribution, deposited or removed modification, and principal function.

Enzyme	Family/Superfamily	Phylogenetic Distribution	Modification and Sequence Context	Principal Function	Section(s)
DIM-2	DNMT1 superfamily (DNMT1-like fold; functions de novo)	Ascomycota; Mucoromycota (Phycomyces)	5mC; all CpN contexts, CpT preference	De novo methylation directed by HP1/H3K9me3; antiviral protection in vegetative hyphae	Section 2.1 and Section 2.3
RID	Masc1/RID subfamily (within the DNMT1 superfamily)	Ascomycota	5mC; in vitro catalysis not detected	Initiates RIP; nucleates euchromatin-to-heterochromatin transition (HDA-1-dependent)	Section 2.2
DNMT1-RFD	DNMT1 maintenance class with replication focus domain	Basidiomycota	5mC; predominantly CpG	Replication-coupled CpG maintenance	Section 2.5
DNMT5	SNF2-ATPase/MTase fusion	Basidiomycota; some Mucoromycota	5mC; CpG (hemimethylated substrate)	ATP-dependent high-fidelity maintenance; centromere integrity	Section 2.4
MTA1c complex: Mta1/Mta9/P1	MT-A70 family	Early-diverging fungi (Mucoromycota)	6mA; symmetric ApT	Symmetric 6mA writer; gene-body localization	Section 4.2
MetB	N6-MTase family	Mucoromycota; some Glomeromycota	6mA; asymmetric AAA-6mA-CA motif	Asymmetric 6mA deposition	Section 4.2
Dmt1	AlkB/ALKBH family dioxygenase	Mucoromycota	6mA → 6hmA in vivo	First genetically validated fungal 6mA demethylase; dimorphism	Section 4.2
CcTet	TET/5mC-oxidase family	Basidiomycota	6mA → 6hmA on dsDNA; also 5mC oxidation	First biochemically characterized fungal 6mA eraser	Section 4.2

## Data Availability

No new data were created or analyzed in this study. Data sharing is not applicable to this article.

## Abbreviations

5mC	5-methylcytosine
6mA	N6-methyladenine
BAH1	Bromo-Adjacent Homology 1 domain
BAH2	Bromo-Adjacent Homology 2 domain
BGC	biosynthetic gene cluster
ChIP-seq	chromatin immunoprecipitation sequencing
DAP-seq	DNA affinity purification sequencing
EDF	early-diverging fungi
H3K36me2	histone H3 lysine 36 dimethylation
H3K9me3	histone H3 lysine 9 trimethylation
HDA-1	histone deacetylase 1
HP1	heterochromatin protein 1
IPD	inter-pulse duration
LC-MS/MS	liquid chromatography–tandem mass spectrometry
LINE	long interspersed nuclear element
LT50	median lethal time
MAC	methylated adenine cluster
MeDIP-seq	methylated DNA immunoprecipitation sequencing
MIP	methylation induced premeiotically
MSAP	methylation-sensitive amplification polymorphism
MTase	methyltransferase
ONT	Oxford Nanopore Technologies
RdDM	RNA-directed DNA methylation
RFD	replication focus domain
RFTS	replication foci targeting sequence
RIP	repeat-induced point mutation
SAH	S-adenosyl-L-homocysteine
SAM	S-adenosyl-L-methionine
SMRT	single-molecule real-time (sequencing)
SNF2	sucrose non-fermenting 2 ATPase domain
ssDNA	single-stranded DNA
TE	transposable element
TET	Ten-Eleven Translocation dioxygenase
TSS	transcription start site
UHRF1	ubiquitin-like with PHD and RING finger domains 1
WGBS	whole-genome bisulfite sequencing
